# Epistemic Trust Is a Critical Success Factor in Psychosomatic Rehabilitation—Results from a Naturalistic Multi-Center Observational Study

**DOI:** 10.3390/jcm13010177

**Published:** 2023-12-28

**Authors:** David Riedl, Hanna Kampling, Johannes Kruse, Tobias Nolte, Karin Labek, Christina Kirchhoff, Vincent Grote, Michael J. Fischer, Alexander Knipel, Astrid Lampe

**Affiliations:** 1 Ludwig Boltzmann Institute for Rehabilitation Research, 1100 Vienna, Austria; 2 University Hospital of Psychiatry II, Department of Psychiatry, Psychotherapy, Psychosomatics and Medical Psychology, Medical University of Innsbruck, 6020 Innsbruck, Austria; 3 Department of Psychosomatic Medicine and Psychotherapy, Justus Liebig University Giessen, 35390 Giessen, Germany; 4 Department for Psychosomatic Medicine and Psychotherapy, Medical Center of the Philipps University Marburg, 35037 Marburg, Germany; 5 Anna Freud National Centre for Children and Families, London N1 9JH, UK; 6 Research Department for Clinical, Educational and Heath Psychology, University College London, London WC1E 6BT, UK; 7 Institute of Psychology, University of Innsbruck, 6020 Innsbruck, Austria; 8 VAMED Rehabilitation Center Kitzbuehel, 6370 Kitzbuehel, Austria; 9 VAMED Rehabilitation Center Oberndorf, 5110 Oberndorf, Austria; 10 VAMED Rehabilitation Center Montafon, 6780 Schruns, Austria

**Keywords:** epistemic trust, psychosomatics, depression, rehabilitation, therapy outcome, critical success factors, psychotherapy

## Abstract

Knowledge about critical success factors underpinning beneficial treatment outcomes in psychosomatic inpatient rehabilitation is scarce. The aim of this study was to evaluate the influence of patients’ epistemic stance in relation to the improvement of psychological distress during rehabilitation. In this naturalistic longitudinal observational study, *n* = 771 patients completed routine assessments for psychological distress (BSI-18), health-related quality of life (HRQOL; WHODAS), and epistemic trust (ETMCQ) before (T1) and after (T2) psychosomatic rehabilitation. Patients were grouped as best, average, and worst responders based on their mean BSI-18 changes during treatment, and their mean change in epistemic trust, mistrust, and credulity was compared using repeated measures analyses of variance (rANOVAs). No associations of performance with sex (*p* = 0.09), age (*p* = 0.11), or relationship status (*p* = 0.58) were found. Best responders reported significantly improved epistemic trust (*p* = 0.001) and reduced epistemic mistrust (*p* < 0.001), whereas worst responders reported a significant increase in epistemic mistrust (*p* < 0.001) and credulity (*p* < 0.001). Average responders did not change for either epistemic trust (*p* = 0.11), mistrust (*p* > 0.99), or credulity (*p* = 0.96). Our results underscore the role of the epistemic stance in psychosomatic and psychotherapeutic treatments. These results help to better understand what might determine psychosomatic rehabilitation outcomes and indicate the role of epistemic trust as a critical success factor.

## 1. Introduction

In order to address the increasing challenge of mental disorders as a major incremental reason for overall symptom burden, incapacity for work, and disability pension, psychosomatic rehabilitation clinics are a well-established in-patient treatment offer in Austria and other Western European countries [1]. Following the holistic approach of psychosomatic medicine, psychosomatic rehabilitation integrates a biopsychosocial understanding and multi-factorial etiology of illnesses, whereby biological, psychological, and social factors interact to cause and maintain mental and physical symptoms and impaired functioning. To account for this, psychosomatic rehabilitation provides an interdisciplinary treatment approach with the aim of reducing pathology as well as increasing functioning and quality of life [2,3,4]. In Austria, psychosomatic rehabilitation is normally regulated by the performance profile of the responsible pension scheme (PVA) and generally provides a 6-week (42-day) inpatient rehabilitation treatment consisting of individual and group psychotherapy, pharmacotherapy, medical examinations, physical training, occupational therapy, nutrition counseling, creative therapies, and social counseling [1,5]. The diagnostic spectrum addressed in psychosomatic rehabilitation is wide with high prevalence and co-occurrence of mood, anxiety, adjustment, somatoform, and personality disorders, thereby representing a similar spectrum as encountered in primary health care [4]. Most patients undergoing psychosomatic rehabilitation have been experiencing a chronic course of mental disorders that is associated with significant limitations in social or occupational participation [6]. The overall effectiveness of psychosomatic medicine in the treatment of mental disorders has been shown across numerous studies [7,8,9,10]. In addition, various barriers and facilitators of treatment success have been identified, including the social environment, the preparation for inpatient care, work, and health care, as well as patient characteristics [11]. However, about a quarter of patients (20–30%) do not respond to the offered treatment and about 10% even deteriorate during their stay [12]. Although personal characteristics—mainly in terms of self-reflection, awareness, or acceptance—have also been identified as relevant patient factors of psychosomatic rehabilitation outcome [11], only very little is known about their role as transpersonal and transdiagnostic critical success factors in psychosomatic rehabilitation.

In a recent study, mentalizing has been identified as a potential patient factor affecting psychosomatic rehabilitation regarding the improvement of psychological distress [13]. Fonagy and Bateman first introduced the concept defined mentalizing as a mental process that facilitates the understanding and representation of intentional mental states in oneself and others by taking into account one’s own thoughts, needs, emotions, wishes, and desires as well as those of others [14,15,16]. Our research indicates that the patients’ epistemic stance may be a fundamental prerequisite for the development of their mentalizing abilities during psychosomatic inpatient rehabilitation [13]. ‘Epistemic stance’ refers to the stance an individual takes with respect to the delivery of social knowledge, namely your relation to yourself and others, both as ‘providers of knowledge’ and ‘receivers of knowledge’ [17]. The epistemic stance comprises epistemic trust as well as its disruptions, namely epistemic mistrust and epistemic credulity, and describes the openness to social learning with the ability to appropriately evaluate whether information from other individuals or sources is considered by the patient as trustworthy, relevant to the self, and generalizable to other contexts [18]. Although epistemic mistrust refers to a tendency to consider all information sources as unreliable or ill-intended, resulting in resistance to being influenced by others, epistemic credulity comprises a lack of vigilance and discrimination between trustworthy and untrustworthy information, making individuals more vulnerable to misinformation and exploitation [19]. The significance of this concept is particularly evident in the medical system, where the patient’s epistemic stance significantly shapes the reception of information provided by their healthcare professional, who serves as the knowledge provider, regarding their disease and its treatment. Specifically, patients with heightened levels of epistemic mistrust may encounter considerable difficulty in placing trust in their treating physician or psychologist, increasing the likelihood of discontinuing or altering their treatment.

The development of both epistemic trust and mentalizing is based on experiences in early childhood facilitated by secure attachments and interpersonal relationships alongside adequate experiences of being sensitively responded to [18,20,21]. However, children growing up in an environment characterized by sustained and high parental distress, unreliable or malevolent caregiving experiences, or adverse childhood experiences are assumed to experience compromised learning about the social world that results in underdevelopment or even breakdown of epistemic trust [22,23]. Mistrust may arise as people’s motives are over-interpreted, their intentions assumed to deviate from those declared, and the source of the information perceived as not respected. In addition, epistemic hypervigilance and epistemic mistrust may propel the individual to reject the content of the information, confuse its meaning, or even misinterpret it as being malignant [24]. Epistemic mistrust leads to misattributed intentions and the assumption of malevolent motives behind others’ actions, fostering epistemic hypervigilance or inappropriate trust in informants misjudged as credible [24].

It has been suggested that many types of psychopathology could indeed be characterized by temporary or permanent disruptions of epistemic trust and the social learning process it enables [24], and that, in turn, psychotherapy might offer a chance to break this vicious cycle of epistemic mistrust and credulity by inducing epistemic trust, and therefore, social learning within and beyond the therapeutic relationship [18,25,26,27]. To our knowledge, there is no research assessing the role of the epistemic stance as a potential critical success factor underpinning the patient’s propensity to be open or closed towards new information and relational experiences in inpatient psychosomatic rehabilitation. Therefore, the present study aims to investigate whether the patients’ treatment performance during rehabilitation is associated with changes in epistemic trust, epistemic mistrust, or epistemic credulity.

## 2. Materials and Methods

### 2.1. Patients and Procedures

Data were collected as part of the clinical routine procedures at the Psychosomatic Rehabilitation Center Montafon (Schruns, Austria) and the Psychosomatic Rehabilitation Center Oberndorf (Oberndorf, Austria). Adult patients underwent multidisciplinary and multimodal inpatient rehabilitation, with costs being covered by the Austrian Social Security Institution. Data were collected in a systematic standardized survey procedure at the beginning (T1; within the first week) and end (T2; within the last week) of the rehabilitation treatment. At the time of the admission, patients were asked whether they were willing to participate in an observational study. Upon written informed consent, they were included. Data were collected electronically using a multifunctional web-based application called the Life App, which is based on the Computer-Based Health Evaluation Software (CHES v7.4) [28]. Patients were included in this study if (a) they completed the BSI-18 assessment at T1 and T2, (b) key sociodemographic data (age, sex) was available, and (c) if ICD-10 diagnosis at admission was given. This study was approved by the Ethics Commission of the University of Innsbruck (no. 75/2023) and was conducted according to the principles of the Declaration of Helsinki.

### 2.2. Psychosomatic Inpatient Rehabilitation Treatment

Rehabilitation lasted six weeks with nine hours of therapeutic units per week. Patients received multidisciplinary and multimodal treatment, which typically included two 90 min sessions of symptom-specific group therapy (e.g., for trauma, burn-out, somatization, pain, etc.), one hour of individual psychotherapy, two hours of group sessions for relaxation training, as well as additional occupational therapy and physiotherapy if necessary. Additionally, each patient participated in one group session to develop medium-term goals and therapy focus for the next week, as well as two hourly group sessions for resource activation. The guidelines of the Austrian Social Security Institution, which require certain frequencies for the respective therapies, served as a basis for the treatment planning so that treatment provision reflects state of the art provision as per guidelines [29].

### 2.3. Measures

#### 2.3.1. Brief Symptom Inventory (BSI-18)

Psychological distress was assessed with the Brief Symptom Inventory (BSI-18) consisting of 18 items. A total score and three subscale scores (depression, anxiety, somatization) can be calculated. Good reliability and validity for the subscales and total scores have been reported [30].

#### 2.3.2. Epistemic Trust, Mistrust, and Credulity Questionnaire (ETMCQ)

The Epistemic Trust, Mistrust, and Credulity Questionnaire (ETMCQ) is used to assess a person’s capability of epistemic trust. It consists of 15 items measuring the three subscales ‘epistemic trust’, ‘mistrust’, and ‘credulity’ resulting in a sum score between 15 and 105. High trust reflects a person’s ability to be open to opportunities for social learning, whereas high mistrust indicates a tendency to treat information sources as unreliable and to avoid being influenced by communication from others. High credulity reflects a person’s lack of clarity about their own position, which can lead to high vulnerability to misinformation and exploitation by others [19]. The ETMCQ has been validated in several languages and cultures [19,31,32]. 

#### 2.3.3. World Health Organization Disability Assessment Scale (WHODAS)

The World Health Organization Disability Assessment Scale (WHODAS 2.0) is a self-report questionnaire used to assess activity and participation limitations in conjunction with the ICF. It consists of six domains of health-related quality of life (HRQOL), namely mobility, cognition, self-care, social functioning, life activities, and participation in the society), which can be summed up to a total score. The WHODAS 2.0 is scored on a continuum from 0 to 100, where 0 indicates the absence of disability in all domains, whereas 100 indicates maximal disability. The WHODAS 2.0 has been identified as a valid and reliable self-report instrument for the assessment of disability [33].

### 2.4. Statistical Procedures

Patients with complete datasets for the BSI-18 at T1 and T2 were included in the analyses. To determine the best, average, and worst responders during the rehabilitation, the mean improvement in BSI-18 total scores was divided into three equal groups. To avoid influence of baseline differences, the ‘performance score (T2D)’ was used instead of the delta. The T2D is a simple solution to account for baseline differences, based on the formula T2 + (T2 − T1). The formula reflects the individual performance and considers the functional status at the beginning of rehabilitation (changes from T1 to T2; Δ) without problems of mathematical coupling or regression effects, as seen in ANCOVA [34]. The association’s baseline variables (sex, age, relationship status, and ICD-10 diagnosis) with the performance during rehabilitation were investigated with χ^2^-tests and analyses of variance (ANOVAs). Additionally, mean group differences in the performance groups regarding the level of HRQOL (WHODAS total score and subscales) at the end of treatment (T2) were analyzed using ANOVAs.

To investigate the association of epistemic trust, mistrust, and credulity with the overall performance during rehabilitation, changes in ETMCQ scores before and at the end of rehabilitation in relation to the BSI-18 performance (best, average, worst performance) were compared using repeated measure ANOVAs with Bonferroni-corrected post hoc analyses. The mean levels of the three ETMCQ subscales served as dependent variables (change from T1 to T2) and the BSI-18 performance groups as grouping variables. Effect size values of *d* < 0.2 and *η*^2^ < 0.01 were considered negligible, *d* ≥ 0.2 and *η^2^* ≥ 0.01 as small effects, *d* ≥ 0.5 and *η*^2^ ≥ 0.06 as medium effects, and *d* ≥ 0.8 and *η*^2^ ≥ 0.14 as large effects [35,36]. A priori sample size calculations indicated that the given ad hoc sample was sufficiently large to detect mean differences in even small effect size (f = 0.1) in the within–between group interaction (three groups, two time points; α = 0.05; 1 − β = 0.95). Estimation of the sample size was conducted using G*Power (3.1) and all other calculations were conducted using IBM SPSS (v21).

## 3. Results

Of the initial *n* = 1065 patients, *n* = 104 (9.8%) patients were excluded due to missing clinical data and sociodemographic data, and another *n* = 190 (19.8%) patients due to missing BSI-18 data. The remaining *n* = 771 patients were included in the analyses. A flow chart is presented in Supplementary Materials. Included and excluded patients did not significantly differ in epistemic trust (*p* = 0.87), epistemic mistrust (*p* = 0.63) and epistemic credulity (*p* = 0.19) at baseline (T1), whereas excluded patients had slightly higher depression (*p* = 0.051), anxiety (*p* = 0.021), and somatization scores (*p* = 0.008). However, effect size calculations indicated that group differences were of negligible effect size (g = 0.14–0.18).

The majority of the included patients were female (60.2%), married (50.8%), and between 50 and 60 years old (45.0%). The most frequent clinician-rated ICD-10 main diagnosis was depressive disorder (62.8%). Based on the patients’ self-reported symptoms (BSI-18), three-quarters of the patients (75.1%) scored above the cut-off for depression, 79.9% for anxiety, and 61.3% for somatization. For details, see Table 1.

### 3.1. Best, Average, and Worst Responders during Psychosomatic Rehabilitation

Based on the T2D scores of the BSI-18, the sample was divided into best (mean T2D = −10.2 ± 8.1 points; *n* = 261, 33.9%), average (mean T2D = 5.0 ± 4.2 points; *n* = 259, 33.6%), and worst responders (mean T2D = 32.0 ± 15.0 points; *n* = 251, 32.6%). In the context of the BSI-18, higher negative T2D scores usually indicate significant improvement. However, as the T2D accounts for the baseline score, patients may not necessarily have negative T2D scores even if they report a substantial reduction in symptoms. For instance, a decrease from 53 points at T1 to 29 points at T2 results in a T2D score of five. This scenario is analogous to a patient with a score of 5 points at T1 and 5 points at T2, where the T2D score would also be five. Better performance was associated with a significantly better overall QoL and functioning at the end of treatment (T2) (F = 216.605; *p* < 0.001; *η*^2^ = 0.38), as well as a better level of cognitive functioning (F = 129.960; *p* < 0.001; *η*^2^ = 0.32), mobility (F = 177.200; *p* < 0.001; *η*^2^ = 0.26), self-care (F = 90.047; *p* < 0.001; *η*^2^ = 0.19), social functioning (F = 129.491; *p* < 0.001; *η*^2^ = 0.26), domestic responsibilities (F = 100.451; *p* < 0.001; *η*^2^ = 0.21), work responsibilities (F = 113.016; *p* < 0.001; *η*^2^ = 0.24), and social participation (F = 155.063; *p* < 0.001; *η*^2^ = 0.29).

**Figure 1:**
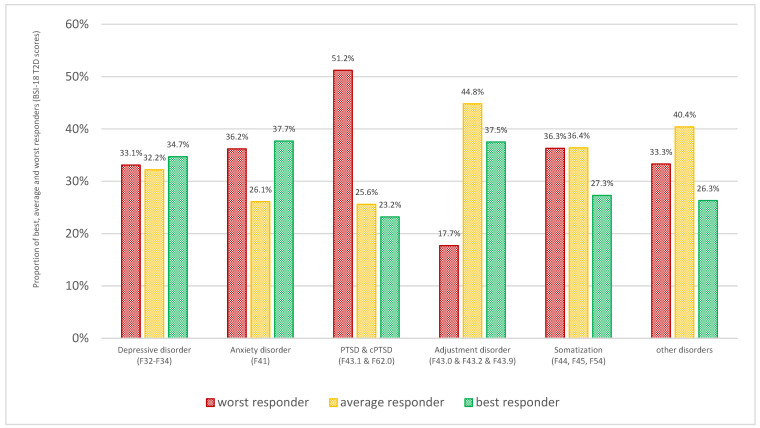
Proportion of best, average, and worst responders based on the T2D scores of the BSI-18, stratified for ICD-10 diagnostic groups.

The performance groups did not statistically differ in terms of sex (χ^2^ = 4.82, *p* = 0.09), age (F(2, 770) = 2.253, *p* = 0.11), and relationship status (χ^2^ = 4.69, *p* = 0.58). However, ICD-10 diagnosis at baseline was significantly associated with performance: patients with trauma-related disorders were classified as worst responders more often than patients with other disorders, whereas patients with adjustment disorders had the lowest proportion of worst responders (χ^2^ = 21.61, *p* = 0.017). For details, see Figure 1.

### 3.2. Epistemic Trust as a Critical Success Factor

**Figure 2:**
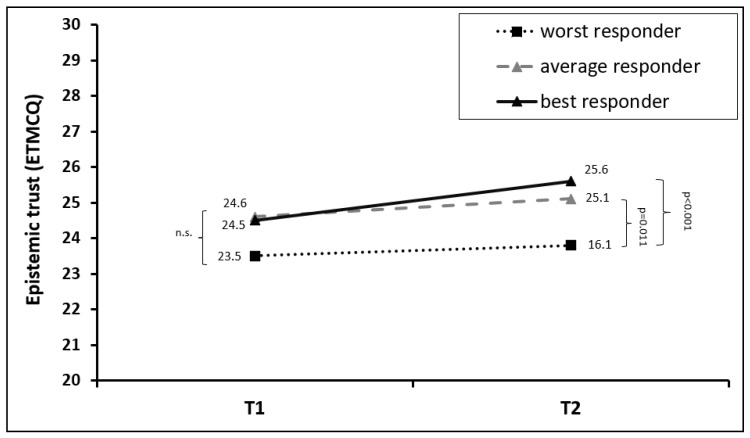
Mean changes in epistemic trust in the first (T1) and last (T2) week of rehabilitation treatment, stratified for best, average, and worst responders in the T2D BSI-18 total score. The abbreviation “n.s.” stands for “not significant”.

In the total sample, an overall improvement in epistemic trust was observed (F = 10.902, *p* = 0.001, *η*^2^ = 0.014). However, this was mainly driven by the improved levels of epistemic trust in the best responder group (F = 11.869, *p* = 0.001, *η*^2^ = 0.015), whereas no significant change was observed for average (F = 2.583, *p* = 0.11, *η*^2^ = 0.003) or worst responders (F = 0.479, *p* = 0.49, *η*^2^ = 0.001). At baseline (T1), no significant differences in the epistemic trust subscale levels were observed between the three groups (*p* = 0.08–0.99). By the end of treatment both best (*p* < 0.001) and average responders (*p* = 0.011) reported significantly higher scores than worst responders. For details, see Figure 2 and Table 2.

**Figure 3:**
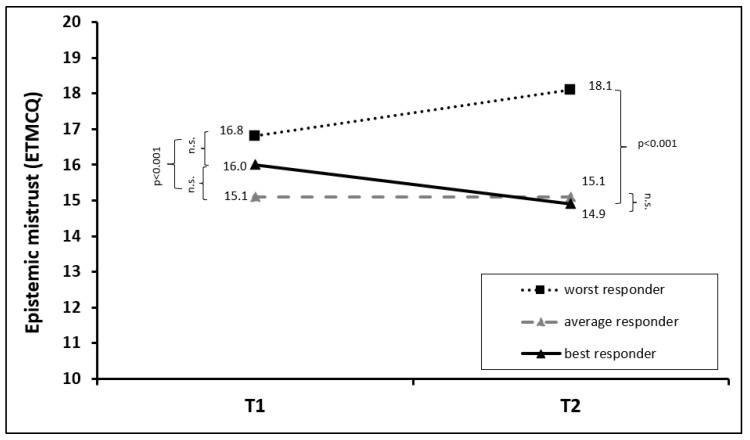
Mean changes in epistemic mistrust in the first (T1) and last (T2) week of rehabilitation treatment, stratified for best, average, and worst responders in the T2D BSI-18 total score. The abbreviation “n.s.” stands for “not significant”.

In contrast, there was no statistically significant change in epistemic mistrust in the total group (F = 0.459, *p* = 0.50, *η*^2^ = 0.001). However, a significant time × group effect was detected (F = 22.225, *p* < 0.001, *η*^2^ = 0.055), indicating differences in the development of epistemic mistrust across the performance group. Post hoc analyses revealed that the level of epistemic mistrust significantly decreased in the group of best responders (F = 17.354, *p* < 0.001, *η*^2^ = 0.022), whereas it increased in the group of worst responders (F = 27.407, *p* < 0.001, *η*^2^ = 0.035). No significant change was observed in average responders (F = 0.000, *p* > 0.99, *η*^2^ = 0.000). At baseline, a significant difference was only found between average and worst responders (*p* < 0.001). Interestingly, the best responders scored between average and worst responders but showed the highest decrease in epistemic mistrust during therapy. At the end of the treatment, the worst responders showed significantly more epistemic mistrust than the average and best responders (both *p* < 0.001). For details, see Figure 3 and Table 2.

**Figure 4:**
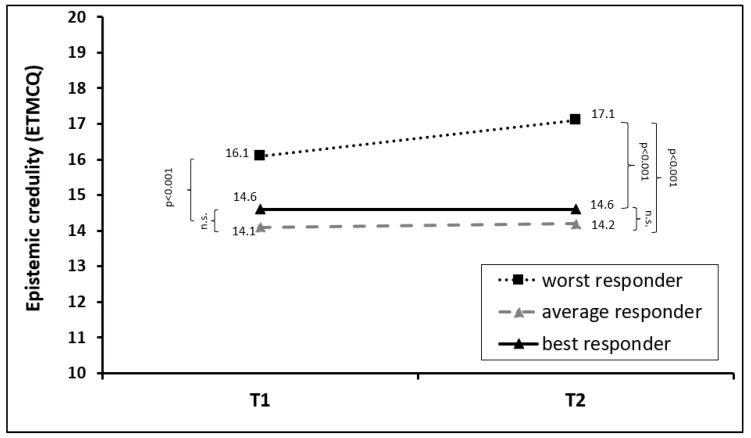
Mean changes in epistemic credulity in the first (T1) and last (T2) week of rehabilitation treatment, stratified for best, average, and worst responders in the T2D BSI-18 total score. The abbreviation “n.s.” stands for “not significant”.

For epistemic credulity, a slightly different pattern was observed: overall, there was an increase in epistemic credulity in the total sample (F = 7.374, *p* = 0.007, *η*^2^ = 0.010). In addition, a significant time * group effect was observed (F = 5.110, *p* = 0.006, *η*^2^ = 0.013), indicating differences in the development of epistemic mistrust across the performance group. Post hoc tests revealed that the overall change was mainly caused by a significant increase in epistemic credulity in the worst responders group (F = 17.048, *p* < 0.001, *η*^2^ = 0.022), whereas no significant change was observed for average (F = 0.225, *p* = 0.64, *η*^2^ = 0.000) and good responders (F = 0.002, *p* = 0.96, *η*^2^ = 0.000). Worst responders started therapy with significantly higher credulity scores than average (*p* < 0.001) and best responders (*p* = 0.001), whereas those two groups did not differ at baseline (*p* = 0.56). A similar pattern was observed at the end of treatment: although average and best responders did not change in the extent of their epistemic credulity, in the group of worst responders the credulity levels even increased during therapy. For details, see Figure 4 and Table 2.

## 4. Discussion

Psychosomatic inpatient rehabilitation is a well-established and effective interdisciplinary treatment approach for a variety of mental disorders in various European countries [7,8,9,10]. However, very little is known about the underlying mechanisms that determine treatment success, non-response, or even worsening of patients’ mental health. After grouping patients into three groups of best, average, and well responders based on the change in psychological symptoms during a six-week multimodal inpatient rehabilitation treatment, our results indicate that common patient factors such as sociodemographic characteristics did not determine performance. However, patients with trauma-related disorders were more likely to show worse treatment outcome.

Based on our previous results which identified the patients’ mentalizing capability and epistemic stance as critical success factors underpinning success in psychosomatic rehabilitation outcomes [13], the current study further examined the role of the epistemic stance with regard to the performance during psychosomatic rehabilitation by considering the three specific facets that shape a person’s disposition towards new learning from others and their openness to new relational experiences: trust, mistrust, and credulity. At the beginning of treatment, we found no significant group differences in either trust, mistrust, or credulity between groups, indicating that the initial epistemic stance did not account for subsequent performance during their rehabilitation inpatient stay. These results also suggest that changes in epistemic stance may be engendered through interpersonal experiences during rehabilitation—within the therapeutic alliances with different professionals involved in treatment but also the wider patient collective. In line with this, we observed differentiated changes in the three performance groups. Patients in the best performance group showed significant increases in epistemic trust and simultaneous decreases in epistemic mistrust, whereas no changes were observed regarding epistemic credulity. For average responders, no significant changes could be found in epistemic trust, mistrust, or credulity. In contrast, the worst responders group showed no significant changes in epistemic trust, whereas both epistemic mistrust and credulity increased significantly. In addition, in comparison to the best and average responders, the worst responders demonstrated significantly higher epistemic mistrust and epistemic credulity as well as epistemic credulity at the end of rehabilitation.

It has been described by Nolte, Hutsebaut, Sharp, Campbell, Fonagy, and Bateman [23] that for patients who are prone to avoid mentalizing others (the ‘we-mode’) due to a protective fear of blending with others’ minds, a mentalizing position may be experienced as a threatening experience. It can be unsettling for those prone to feeling overwhelmed or overshadowed by others’ thoughts (epistemic credulity), those who avoid connections (epistemic mistrust), or those swaying between both positions in a disorganized fashion. Hence, it makes sense that patients who showed unfavorable outcomes of psychosomatic rehabilitation might also have experienced a worsening of epistemic mistrust and epistemic gullibility. The mentalization-promoting environment of the psychotherapeutic interventions might have overwhelmed the individual and thus led to a worsening of the epistemic stance.

Our results indicate that changes in the epistemic stance might be directly associated with the performance during psychosomatic rehabilitation. These results can help to better understand the relationship between the epistemic stance and psychopathology. Recent research has shown that mentalizing and epistemic vigilance were associated with an increased risk of acting out behaviors as well as internalizing and externalizing problems in adolescents [37,38] and that epistemic mistrust was associated with an increased risk of developing depressive disorders among young adults during the COVID-19 pandemic [39], whereas robust levels of epistemic trust and mentalizing were identified as protective factors for emotional dysregulation of adolescents during the COVID-19 lockdowns [40]. Research has further suggested that psychopathologies are associated with an impaired epistemic stance and alongside this the disrupted social learning process it enables [24]. Two recent studies demonstrated that epistemic disruption was directly associated with trauma-related disorders [41] and that restoring epistemic trust as well as reductions in epistemic mistrust were linked to improved trauma-related symptoms [42]. Hence, the epistemic stance might prove to be a key component of rehabilitation outcomes. Psychotherapy has been suggested as a means to break the vicious cycle of epistemic mistrust and credulity by inducing epistemic trust [18,25,26,43,44]. Following this line of thinking, psychosomatic rehabilitation might offer an opportunity to use the psychotherapeutic context—including the therapeutic alliance—to its advantage by fostering interpersonal experiences that increase mentalizing and thus build epistemic trust and reduce epistemic mistrust via shared understanding and interpersonal experiences in the so-called ‘we mode’ [23,45]. Fonagy and colleagues highlight that cooperative social learning and the development of epistemic trust need to occur within and beyond the therapeutic relationship [26]. The specific environment of inpatient psychosomatic rehabilitation with its interdisciplinary team and the availability of interactions with other patients offers a direct and safe training ground for a better understanding of self and others. These experiences may be transferred into the broader and thus engendering sustained salutogenetic feedback loops [45]. The importance of trust and the psychotherapeutic relationship becomes even more evident when considering that a dysfunctional—and possibly not trusting—therapeutic alliance has been associated with adverse outcomes during psychosomatic rehabilitation [12,46]. Next to its many advantages, psychosomatic rehabilitation often requires a socio-medical evaluation of patients’ working ability and level of functioning during their stay. This evaluating character might be experienced as challenging when it comes to establishing trust in therapists and physicians. Knowing about the importance of epistemic trust and mistrust might help clinicians better understand possible disruptions during a patient’s treatment by (i) bearing in mind potential causes that might enhance mistrust, (ii) more directly exploring the individual nature of such epistemic dynamics with every patient (e.g., during treatment: ‘What could I do to help us understand better when you feel you cannot trust me?’ or ‘Could you help me spot when I may have said or done something that could undermine your trust in me or the team?’), (iii) communicating to the patient that these difficulties are being recognized, and (iv), as a result, establishing a joint focus for both patient and therapist to come back to this understanding when a rupture occurs. In addition, the facilitation of not only a good but also trustworthy psychotherapeutic relationship alongside encouragement to focus more on positive experiences outside of this relationship might further enhance rehabilitation outcomes. Future research needs to examine this and should consider the epistemic stance as a potential success factor for psychosomatic inpatient rehabilitation while also establishing whether the epistemic stance adds incremental validity in relation to other constructs such as mentalizing, paranoia, or traditional assessments of the therapeutic alliance, all of which are associated with treatment outcome.

### Strength and Limitations

This study has several strengths and limitations. To our knowledge, this is the first study to investigate the role of the epistemic stance as a potential factor in explaining therapy outcomes in psychosomatic rehabilitation. The observational design of this study, however, limits the causal interpretation of the study results. Nevertheless, since the data were assessed in clinical routine without a narrow selection of patients, we consider the results of our study to be representative of patients in inpatient rehabilitation treatments. This is further underscored by the similarity of results compared to other studies in the psychosomatic rehabilitation setting [47]. Additionally, a recent study has shown that therapy outcomes in the psychosomatic rehabilitation setting based on real-world data collected from relatively unselected samples during clinical routine may substantially differ from laboratory randomized controlled trials [48] and thus highlight the importance of observational real-world outcome studies.

## 5. Conclusions

Our results add to the growing body of evidence regarding the role of the epistemic stance in psychopathology as well as psychosomatic and psychotherapeutic treatments. We demonstrate that good performance during psychosomatic rehabilitation is accompanied by improvements in epistemic trust, whereas conversely, worst performance during psychosomatic rehabilitation is associated with increasing epistemic mistrust and credulity. These results help us better understand what might determine psychosomatic rehabilitation outcomes and indicate the role of epistemic trust as a critical success factor. 

## Figures and Tables

**Table 1:** Sociodemographic data (*N* = 771).

	*N*	%
Mean age (SD)		
<40	179	(23.2)
40–50	191	(24.8)
50–60	347	(45.0)
>60	54	(7.0)
Sex		
female	464	(60.2)
male	307	(39.8)
Relationship status		
Divorced	112	(14.5)
Single	253	(32.8)
Married	392	(50.8)
Widowed	12	(1.6)
Missing	2	(0.3)
ICD-10 diagnosis ^1^		
Depressive disorder (F32–F34)	484	(62.8)
Anxiety disorder (F41)	69	(8.9)
PTSD/cPTSD (F43.1/F62.0)	43	(5.6)
Adjustment disorder (F43.0, F43.2, F43.9)	96	(12.5)
Somatization disorder (F44, F45, F54)	22	(2.9)
Other disorder	57	(7.4)
BSI-18		
Depression	579	(75.1%)
Anxiety	616	(79.9%)
Somatization	473	(61.3%)

SD = standard deviation; PTSD = post-traumatic stress disorder; cPTSD = complex post-traumatic stress disorder; ^1^ patients may have multiple diagnoses; thus, the cumulative number of diagnoses may exceed the total number of patients.

**Table 2:** Mean BSI-18 scores in the first (T1) and last (T2) week of rehabilitation, stratified across treatment performance on the BSI-18 (T2D).

BSI-18 Performance ^1^		T1		Post Hoc Tests T1			T2		Post Hoc Tests T2			Time		Group		Time × Group	
		M	(SD)	W-A	W-B	A-B	M	(SD)	W-A	W-B	A-B	*p*	*η* ^2^	*p*	*η* ^2^	*p*	*η* ^2^
Epistemic trust	worst	23.5	5.2				23.8	5.5				0.49	<0.01				
	average	24.6	4.8				25.1	5.2				0.11	<0.01				
	best	24.5	5.5				25.6	4.4				0.001	0.015				
	total	24.2	5.2	0.08	0.10	1.0	24.8	5.1	0.011	<0.001	0.74	0.001	0.014	0.001	0.018	0.15	<0.01
Epistemic mistrust	worst	16.8	4.4				18.1	4.0				<0.001	0.04				
	average	15.1	4.5				15.1	4.4				1.00	<0.01				
	best	16.0	4.5				14.9	4.3				<0.001	0.02				
	total	15.9	4.5	<0.001	0.13	0.07	16.0	4.5	<0.001	<0.001	1.00	0.50	<0.01	<0.001	0.067	<0.001	0.06
Epistemic credulity	worst	16.1	4.9				17.1	5.0				<0.001	0.02				
	average	14.1	4.2				14.2	4.3				0.64	<0.01				
	best	14.6	4.8				14.6	4.5				0.96	<0.01				
	total	14.9	4.7	<0.001	0.001	0.56	15.3	4.8	<0.001	<0.001	0.84	0.007	0.010	<0.001	0.057	0.006	0.01

T1 = within the first week of rehabilitation treatment; T2 = within the last week of the rehabilitation treatment; ^1^ T2D BSI-18 scores (i.e., T2 + (T2 − T1)) were divided into three equal groups: best (B), average (A), and worst (W) responders.

## Data Availability

The datasets analyzed in this manuscript are not publicly available due to ethical and legal restrictions. If not already reported within this work, the authors may provide descriptive data on individual medical indicators for admission and discharge or the expected change due to inpatient health care for various groups and diagnoses. Requests for access to anonymized datasets should be directed to the corresponding author (david.riedl@rehabilitation.lbg.ac.at).

## Supplementary Materials

The following supporting information can be downloaded at: https://www.mdpi.com/article/10.3390/jcm13010177/s1, Figure S1: Flow chart for study sample.
