# Supplementary figures and images for: Epistemic Trust Is a Critical Success Factor in Psychosomatic Rehabilitation—Results from a Naturalistic Multi-Center Observational Study
Source: J Clin Med. 2023 Dec 28;13(1):177. doi: 10.3390/jcm13010177 (PMC10780285; doi:10.3390/jcm13010177)

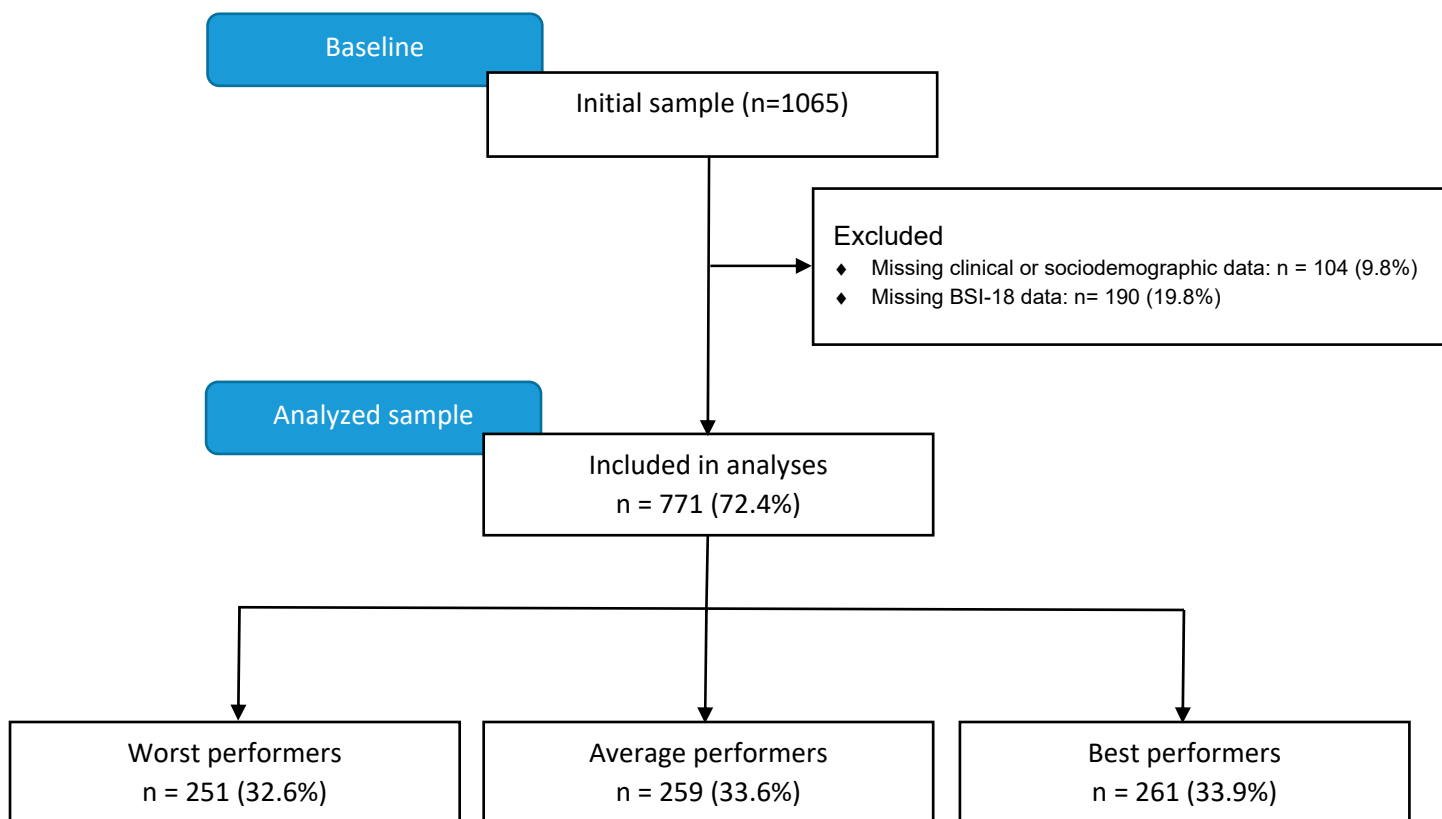

Supplement: Supplementary file 1 [file jcm-13-00177-s001.zip › jcm-2774667-supplementary.pdf]
